# Physically-based in silico light sheet microscopy for visualizing fluorescent brain models

**DOI:** 10.1186/1471-2105-16-S11-S8

**Published:** 2015-08-13

**Authors:** Marwan Abdellah, Ahmet Bilgili, Stefan Eilemann, Henry Markram, Felix Schürmann

**Affiliations:** 1 Blue Brain Project (BBP), École Polytechnique Fédérale de Lausanne (EPFL), Biotech Campus, Chemin des Mines 9, 1202 Geneva, Switzerland

**Keywords:** Modelling and Simulation, Light Sheet Fluorescence Microscopy, Fluorescence Rendering and Visualization, Neocortical Brain Models, In Silico Neuroscience

## Abstract

**Background:**

We present a physically-based computational model of the light sheet fluorescence microscope (LSFM). Based on Monte Carlo ray tracing and geometric optics, our method simulates the operational aspects and image formation process of the LSFM. This simulated, *in silico *LSFM creates synthetic images of digital fluorescent specimens that can resemble those generated by a real LSFM, as opposed to established visualization methods producing visually-plausible images. We also propose an accurate fluorescence rendering model which takes into account the intrinsic characteristics of fluorescent dyes to simulate the light interaction with fluorescent biological specimen.

**Results:**

We demonstrate first results of our visualization pipeline to a simplified brain tissue model reconstructed from the somatosensory cortex of a young rat. The modeling aspects of the LSFM units are qualitatively analysed, and the results of the fluorescence model were quantitatively validated against the fluorescence brightness equation and characteristic emission spectra of different fluorescent dyes.

**AMS subject classification:**

Modelling and simulation

## Background

Light sheet fluorescence microscopy is a significant non-destructive imaging technique with growing importance for neurobiology. It is used to reconstruct and build detailed three-dimensional atlases of entire brains at cellular resolution, see e.g. [1] for application to a zebrafish brain. Compared to widefield fluorescence or confocal microscopes, this technology is capable of scanning thick transparent tissue samples tagged with fluorescent substances with minimal damaging effects such as phototoxicity and photobleaching [2,3]. The recent advancements in LSFM techniques have turned it out to be an extremely convenient tool for optical sectioning large and clarified specimens to reconstruct their structural aspects [4,5].

Whole brain datasets at cellular resolution open novel avenues for quantitative analysis and provide valuable input to detailed computational modeling of brain tissue. A good understanding of the microscopy optical components and the fluorescence effects is crucial for properly interpreting the resulting images. Once the optical path is understood and the physics of the light passage and fluorescence are carefully modeled, it is possible to go one step further: starting from a computational brain tissue specimen one can forward compute the resulting images of a simulated LSFM. Such an *in silico *[6] microscope using physics simulations is potentially useful in neurobiology for accurate validation of computational brain models against biological data measured in the wet laboratory. Moreover, this system can have significant application in other research fields such as microscopy and image processing. For instance, it can be employed as a virtual imaging platform to predict the performance of different microscopic setups for given experimental conditions. Furthermore, it can be utilized to systematically assess the quality of automated image processing algorithms and workflows used for quantitative analysis.

We propose a computational model of the LSFM to generate unbiased physically-plausible fluorescent images adhering to the *energy conservation law*, aiming to making them comparable to those produced by the actual imaging system. Using Monte Carlo ray tracing and physical principles of geometric optics, our model simulates the image formation process in the LSFM including its main components: the illumination system, the acquisition system and light interaction with fluorescent volumetric models that reflect the content of real biological specimens.

We also introduce a new model for rendering fluorescent volumes using the intrinsic characteristics of fluorescent dyes and then evaluate the accuracy of this model in comparison to the fluorescence brightness equation (FBE) and the emission spectra measured in the laboratory.

### Related models in computational microscopy

Although there are no previous reported investigations for simulating the imaging pipeline of the LSFM, several research studies have presented other computational simulators for creating synthetic images produced by similar microscopes. The models developed in these studies can be classified using Ferwerda's definition of realism in synthetic image generation into *physically-plausible *and *visually-plausible *models [7].

To model an optical microscope on a physically-plausible basis, it is necessary to simulate the main light phenomena that contribute to the image formation process such as absorption, scattering, reflection, refraction, diffraction and fluorescence. This simulation computes the amount of light being detected by the imaging sensors of the microscope relying on the principles of conservation of energy. In contrast, visually plausibility can be achieved by creating a synthetic image that has the same visual appearance of the real one using statistical shape modeling, color manipulation, and image processing filters with controllable parameters. For example, visual plausibility of fluorescence can be achieved by modifying the colors of a non-fluorescent object to mimic an image produced by a fluorescence microscope. In turn, visually-plausible microscopy models cannot be reliably used for systematically validating an in-silico tissue construction process. Nevertheless, they can be very helpful for evaluating the quality of automated post-processing workflows that are used for the analysis of various image stacks produced by different kinds of microscopes.

Svoboda *et al*. followed this approach and presented a multi-stage visually-plausible model for simulating the image acquisition process of the conventional fluorescence microscope [8]. This model was used to assess the performance of their automated segmentation techniques that have been developed to analyse realistic fluorescent image stacks. Lehmussola *et al*. designed a computational framework for simulating microscopic fluorescent images of cell populations [9,10]. This framework was developed to compare the performance of several analysis methods for automated image cytometry. A similar workflow has been presented by Malm *et al*. to simulate the bright-field microscope [11]. It was used to generate synthetic cervical smears images to validate the analysis of large-scale screening algorithms of cervical cancer and mammography images.

Building computational models for simulating microscopic optical pipelines on a physical basis is relatively complex and requires a lot of design and implementation considerations. Kagalwala *et al*. developed a computational model of the image formation process of the differential interference contrast (DIC) microscope that can simulate the variations of the phase of the light waves transmitted through the specimen [12-14]. They used polarized ray tracing [15] and approximations of the diffraction artifacts to compute the light propagation through the specimen and the optical elements of the microscope, presenting a first step to combine the concepts of computer graphics and physics for serving computational biology. This model was applied later to reconstruct the optical properties of unknown three-dimensional biological specimen [16-18]. Preza *et al*. proposed another imaging model of the DIC microscope under partially-coherent illumination [19]. Dye *et al*. presented a similar ray-tracing-based model to simulate the imaging of three-dimensional translucent specimen lit with incoherent light [20]. Their model was also used to address the inverse problem of reconstructing the characteristics of unknown volumetric specimen. Based on the same principle, Sierra *et al*. presented a simplified model of phase propagation within a transparent specimen using the point spread function (PSF) to represent the optical transmission response of the phase contrast microscope (PCM) [21]. Tanev *et al*. presented another model for the PCM based on finite-difference time-domain simulation and a realistic three-dimensional model of the biological cell [22].

In principle, fluorescence microscopes can be modeled relying on the same methods described by Kagalwala [12] and Dye [20], however, due to the absence of convenient and intuitive mathematical models of fluorescence in computer graphics, there is no existence for physically-based models for the fluorescence microscope in general and the LSFM in particular.

The following section briefly reviews the existing fluorescence modeling contributions in computer graphics to date. In the methods section, we explain the formalism of these models and their limitations that motivated us to develop and present our fluorescence model and the in silico LSFM in consequence.

### Prior work in fluorescence modelling

There are numerous research studies in computer graphics that simulate light transport in participating media [23]. The majority of these studies modeled several light phenomena that are interpreted by the ray theory of light such as absorption, scattering, reflection and refraction. We found no deep investigations of modeling fluorescence in the literature. Fluorescence was ignored for several reasons, including its little practical value for rendering natural scenes, and the absence of convenient spectral rendering frameworks that could handle fluorescence efficiently [24,25].

Glassner presented the first steps towards a correct formulation of the rendering equation to account for fluorescence emission in participating media [26]. The formalism of this model has not reflected the distinct properties of the fluorescent media. Cerezo *et al*. [27,28] and Gutierrez *et al*. [29,30] developed further extensions to Glassner's model to account for these properties for the purpose of rendering the fluorescent pigments in the ocean. These extensions were limited in two aspects: they ignored the actual spectral profiles of the fluorescent materials, and they were not validated against theoretical laws nor experimental measurements. Our fluorescence extension is presented to fill this gap.

Other extensions for Glassner's model have been formulated to treat the fluorescence as a surface phenomenon using re-radiation matrices [24,31-34], but the discussion of these models is out of scope.

## Methods (LSFM modeling)

### LSFM description

**Figure 1:**
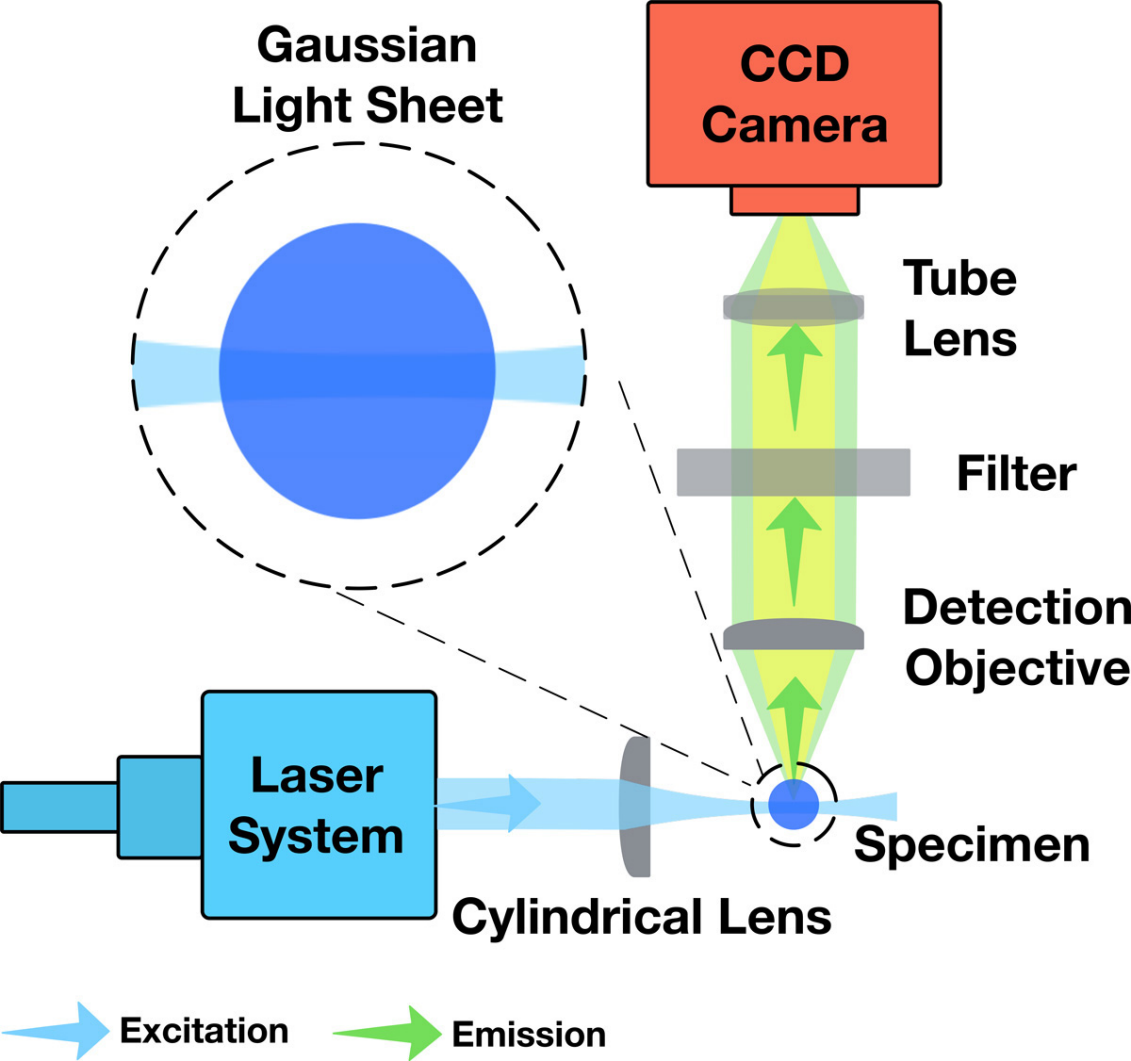
**A simplified top view diagram of the LSFM showing its main components and relative optical setup**. The close up highlights the approximate rectangular profile of a Gaussian sheet intersection with the specimen.

An optimal LSFM generates a stack of fluorescent optical sections from a clarified brain tissue using a fixed thin sheet of light that intersects a moving specimen. Fluorescence excitation and detection are split into two perpendicular and decoupled light paths. The illumination plane excites the fluorescent targets within the specimen. The emitted fluorescence is collected by an objective lens (detection objective) and projected to the charged couple device (CCD) camera of the detection unit using a tube lens. The LSFM generates high axial resolution optical sections because the illumination unit is synchronized with the acquisition unit, aligning the light sheet with the focal plane of the detection objective. A set of filter cubes is installed between the CCD and the detection objective to eliminate the light contributions caused by elastic scattering [2]. A top view diagram of the main components and bench setup of the LSFM is shown in Figure 1.

A physically-plausible simulation of the LSFM requires accurate modelling of the illumination and acquisition stages of the system in addition to modeling the light interaction with fluorescent-tagged specimen volumetric models. Based on geometric optics and the laws of conservation of energy, our simulation uses Monte Carlo ray tracing to compute a fluorescent image that accurately reflects the light propagation in the LSFM. The model takes into consideration the basic optical setup and operational aspects of the microscope as well as the light-specimen interaction. However, the current state of the model ignores wave effects, mainly diffraction, interference and polarization.

### Illumination unit model

The core of the illumination system guides a single-wavelength laser beam into the optical path of the LSFM via a set of optical elements including mirrors, beam splitters, and optical fibers. Using a beam expander, cylindrical lens (typically with a focal length = 150 mm), and an objective lens (illumination objective), this illumination unit expands the input laser beam into a thin light plane (typically 2 - 15 *µ*m thick) that is aligned with the focal plane of the objective lens in the detection unit. Although the light sheet can be produced without the illumination objective, the presence of this element is crucial to remove the aberrations caused by the cylindrical lens, and thus improving the quality of the generated light sheet.

The simulation of the entire illumination unit on an element-by-element basis starting from the laser source and until the generation of the light sheet is computationally expensive and practically infeasible. An alternative way for performing this simulation is the direct modeling of the different aspects of the resulting light sheet and ignoring the complexity of its underlying generation mechanisms. These aspects include its spatial extent, geometric profile, power distribution, and wavelength. Traditional LSFMs use Gaussian beam illumination to excite the specimen. The geometry, illumination profiles, and field of view (FOV) of the Gaussian light sheet depend on the diameter of the input laser beam and the numeric aperture (NA) of the illumination objective. This sheet has a hyperbolic light profile with a Gaussian intensity distribution that is perpendicular to the propagation direction. A fundamental limitation of this illumination scheme is the rapid divergence of the beam (edges are 41% thicker than the beam waist) that prevents the creation of a uniform thin light sheet with large FOV, see Figure 1. The object positioned within the area of the beam waist is sectioned by a light sheet of almost a constant thickness. Consequently, a focused Gaussian beam can be fairly approximated by a rectangular profile [35]. This issue was resolved in advanced LSFMs that have replaced the Gaussian illumination with propagation-invariant Bessel [36] and Airy [37] beams that can yield the same axial resolution and tenfold larger FOV.

Based on the approximation of the Gaussian beam, we have modeled the light sheet by a thin rectangular directional area light with uniform illumination intensity profile and a single excitation wavelength. This model does not have any constraints for LSFMs with Airy and Bessel illuminations, but it is only valid for Gaussian beams when the illumination objective has high NA and small FOV. However, if the lateral dimension of the specimen is relatively small, the approximation of the light sheet model is still effective [38].

### Modelling light-specimen interaction

When the illumination plane excites the different fluorescent targets in the specimen, they emit *isotropically *in all the directions. The spectral power distribution (SPD) of the emitted fluorescence does not only depend on the power of the exciting light sheet, but it also depends on the intrinsic properties of the fluorescent materials (fluorophores) attached to these targets, the concentration of the fluorophores in the tissue, and the wavelength of the exiting laser beam [39].

A physically-based simulation of the interaction between the light and fluorescent specimen model is subject to the existence of a rendering system capable of handling inelastic volume scattering events. Furthermore, it has to accurately calculate the emission SPD profiles in terms of the parameters of the input laser beam and fluorophores embedded in tissue.

We have developed an extension to Glassner's fluorescence model to account for the intrinsic characteristics of the fluorophores including their excitation and emission spectra, extinction coefficients and quantum yield. This extension, discussed in the following section, does not account for quenching nor photobleaching. Table 1 gives a summary of all the relevant terms used in this article.

**Table 1:** Summary for all the important symbols appeared in the text

λ_ex_	Excitation wavelength
λ	Emission wavelength
p	Point in the 3D space
p_s_	Point on the detector surface
*ω*	Incoming direction
*ω′*	Scattering direction
*S*	Source term of the RTE
*f*_ex_	Fluorophore excitation spectrum
*f*_em_	Fluorophore emission spectrum
*L*_ve_	Radiance emitted from point p
*L*_i_	Incoming radiance to point p
*I*	Light flux
*I_ϕA_*	Light flux density
*σ*_s_	Scattering coefficient
*σ*	Absorption cross section
*p*	Phase function
*ϕ*	Quantum yield

### Our fluorescene extension

**Figure 2:**
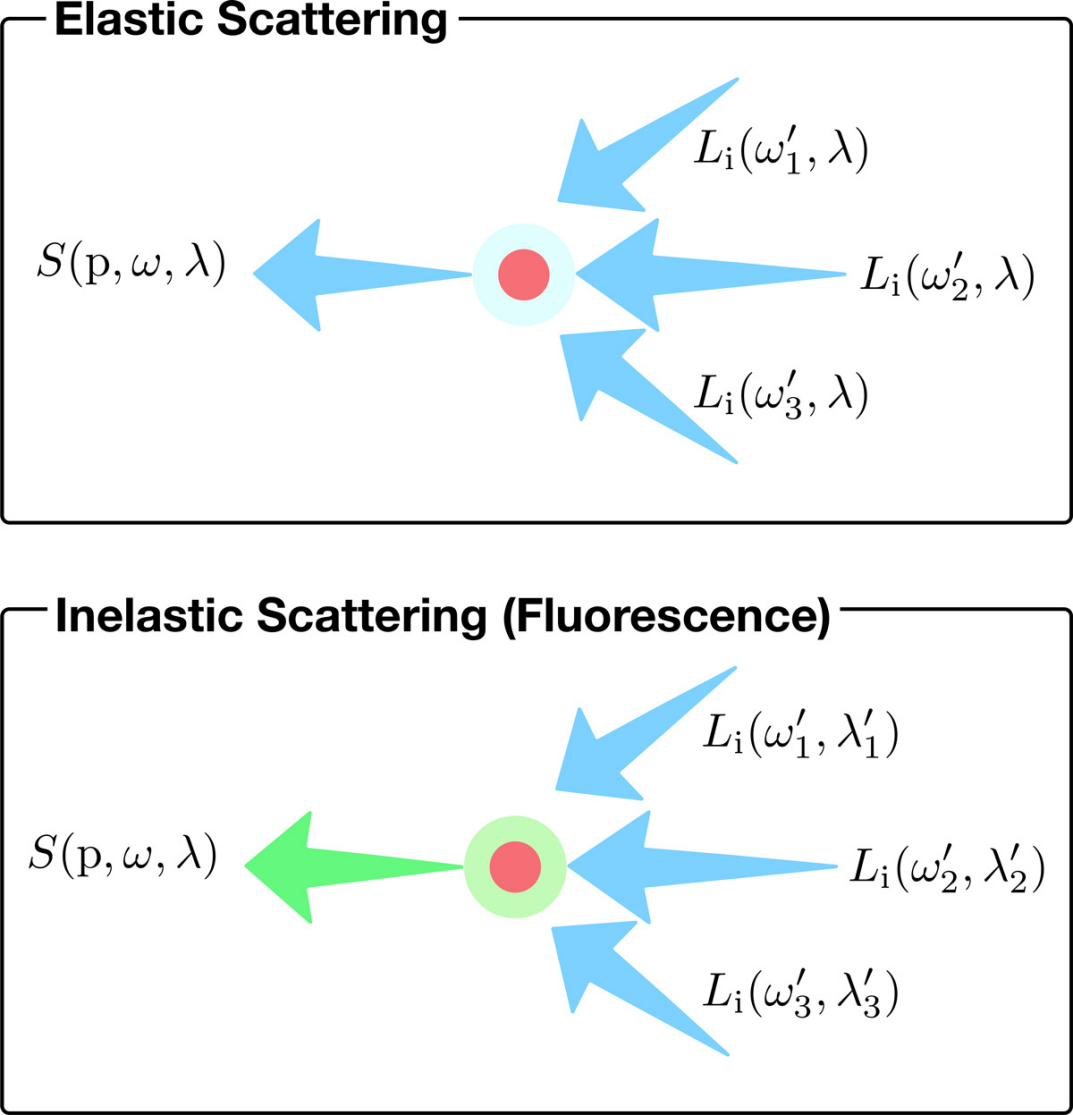
**Simple representation for elastic and inelastic scattering events at a point p**.

The radiative transfer equation (RTE) governs the transfer of energy in the participating media [25]. The source term *S*(p, *ω*) of the RTE is defined by Equation (1) where *L*_ve _is the self-emitted radiance at direction *ω, σ_s _*and *p *are the scattering coefficient and phase function of the medium respectively, and *L*_i_(p, *ω′*) is the incoming radiance from direction *ω′ *at point p, see Figure 2.

(1) $$S\left(\text{p},\;\omega \right)=L_{\text{ve}}\;\left(\text{p},\;\omega \right)\;+\left[\sigma_{s}\left(\text{p},\;\omega \right)\int_{\Omega_{4\pi }}p\left(\text{p},\;\omega^{\prime },\;\omega \right)\;L_{i}\;\left(\text{p},\;\omega^{\prime }\right)\;\text{d}\omega^{\prime }\right]$$

This equation takes into account emission, absorption, and elastic scattering events only. Glassner has extended this equation to account for inelastic scattering by adding a term called fluorescence efficiency *P_f _*(p, λ *← *λ_ex_) that reflects the energy redistribution for each emission-excitation wavelength pair and an integration of the radiance over all the visible wavelengths of the spectrum $R_{\text{v}}$[26]. His extension was limited to the correct formulation of the full radiative transfer equation (FRTE), shown in Equation (2), but he did not give enough elaboration on the fluorescence efficiency term.

(2) $$\begin{array}{c} \;\;\;S\left(\text{p},\omega ,\lambda \right)=L_{\text{ve}}\left(\text{p},\omega ,\lambda \right)+\\ {}[\sigma_{s}\left(\text{p},\omega \right)\int_{R_{\text{v}}}\int_{\Omega_{4\pi }}p\left(\text{p},\omega^{\prime },\omega \right)P_{f}\left(\text{p},\lambda \leftarrow \lambda_{\text{ex}}\right)\\ \;\;\;L_{\text{i}}\left(\text{p},\omega^{\prime },\lambda_{\text{ex}}\right)\;\text{d}\omega^{\prime }\;\text{d}\lambda_{\text{ex}}] \end{array}$$

An extensive discussion of this term was presented later by Cerezo *et al*. [27,28] and Gutierrez *et al*. [29,30] to simulate the inelastic scattering of ocean waters. In this discussion, the fluorescence efficiency term was redefined as the wavelength redistribution function *f_p_*(λ_ex_, λ) that represents the efficiency of the energy transfer between the different wavelengths in terms of the excitation λ_ex _and emission wavelengths λ. This function, Equation (3), was expressed by an absorption function *g_p_*(λ_ex_), a fluorescence emission function *h_p_*(λ), the quantum yield *ϕ*(p), and the wavelength pair.

(3) $$f_{p}\left(\lambda_{\text{ex}},\;\lambda \right)=g_{p}\left(\lambda_{\text{ex}}\right)\;h_{p}\left(\lambda \right)\;\phi \left(p\right)\;\frac{\lambda_{\text{ex}}}{\lambda }$$

The absorption function was assumed to be a binary response that is equal to 1 only if 370 *<*λ_ex _*<*690 and zero otherwise. Moreover, the fluorescence emission function *h_p_*(λ) was oversimplified by the Gaussian function shown in Equation (5), where λ_0 _is the maximum emission wavelength and λ*_σ _*is the wavelength standard deviation.

(4) $$g_{p}\left(\lambda_{\text{ex}}\right)\equiv \left\{\begin{array}{cc} 1, & \text{if}\;370\;<\;\lambda_{\text{ex}}\;<\;690\\ 0, & \text{otherwise} \end{array}\right)$$

(5) $$h_{p}\left(\lambda \right)=\frac{1}{\sqrt{2\pi }\lambda_{\sigma }}\;\text{exp}\;-\frac{{\left(\lambda -\lambda_{0}\right)}^{2}}{2{\left(\lambda_{\sigma }\right)}^{2}}$$

In fact, this model is not valid to accurately express the fluorescence emission in terms of the spectral characteristics of the fluorescent material used in a real experiment. Our extension is presented in Equation (6) to overcome this limit. The energy transfer from excitation wavelength λ_ex _to the emission wavelength λ is primarily determined by the relative contribution of the excitation spectrum *f*_ex _at λ_ex_. The emission power at λ is scaled by the emission spectrum *f*_em _at λ and the quantum yield *ϕ *of the material. Finally, due to the isotropic emission, the phase function of the inelastic term is substituted by 1/4n. Putting all the terms together, our extended fluorescence model can be described by Equation (7), where the term *F *(p) is the binary fluorescence function that is equal to 1 if the point p is fluorescent and 0 otherwise. This equation is the basis of our simulation of the light sheet interaction with the fluorescent tissue models of the specimen in our LSFM model.

(6) $$P_{f}\left(\text{p},\;\lambda \leftarrow \lambda_{\text{ex}}\right)=f_{\text{ex}}\left(\text{p},\;\lambda_{\text{ex}}\right)f_{\text{em}}\left(\text{p},\;\lambda \right)\;\phi \left(\text{p}\right)$$

(7) $$\begin{array}{c} S\left(\text{p},\;\omega ,\;\lambda \right)=L_{\text{ve}}\left(\text{p},\;\omega ,\;\lambda \right)+\\ {}[\frac{1}{4\pi }\;\sigma_{s}\left(\text{p},\;\omega \right)\;\int_{R_{v}}\int_{\Omega_{4\pi }}f_{\text{em}}\left(\text{p},\;\lambda \right)\;f_{\text{ex}}\left(\text{p},\;\lambda_{\text{ex}}\right)\;\phi \left(\text{p}\right)\\ L_{\text{i}}\left(p,\;\omega^{\prime },\;\lambda_{\text{ex}}\right)\;\text{d}\omega^{\prime }\;\text{d}\lambda_{\text{ex}}]\;\times \;F\left(\text{p}\right)+\\ \left[\sigma_{s}\left(\text{p},\;\omega \right)\;\int_{\Omega_{4\pi }}p\left(\text{p},\;\omega^{\prime },\;\omega \right)\;L_{\text{i}}\left(\text{p},\;\omega^{\prime }\right)\;\text{d}\omega^{\prime }\right]\;\times \;\left[1-F\left(\text{p}\right)\right] \end{array}$$

### Acquisition system model

**Figure 3:**
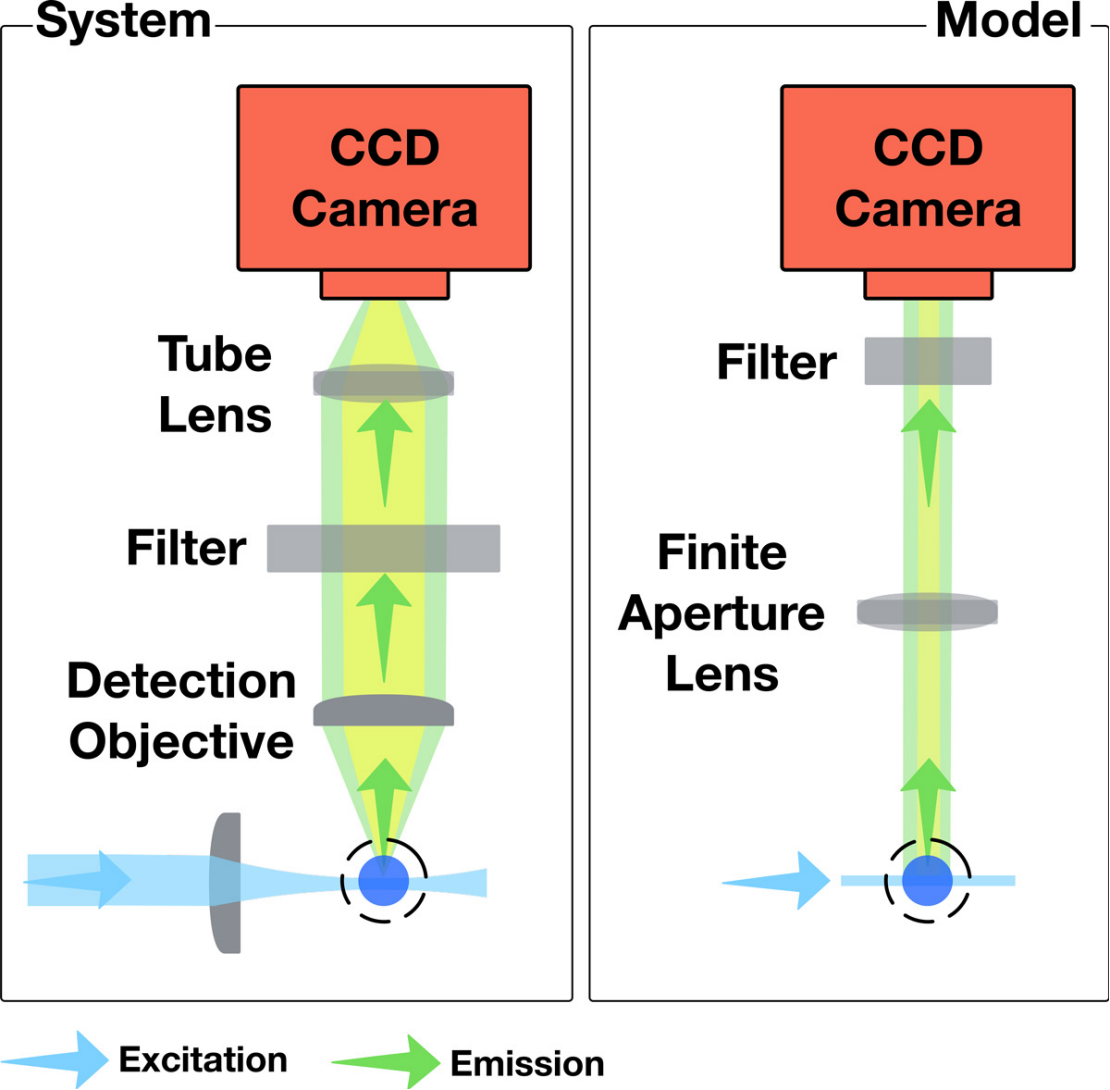
**Comparison between the acquisition system of the LSFM and our model**. The coupling between the detection objective lens and the tube lens is modeled by an orthographic camera.

There are two main lenses in the acquisition unit: the detection objective that collects the emitted fluorescence from the specimen across the entire FOV, and an infinity-corrected tube lens that projects the intermediate image on the CCD. The coupling between the two lenses form a telecentric lens system that produces an orthographic view of the acquired optical section. As shown in Figure 3, the acquisition unit can be modeled by a thin lens orthographic camera with finite aperture to simulate the depth of field effects [40,25]. This model provides an accurate simulation of the image formation process in the real microscope, however, its performance is subject to either high sampling rates or importance sampling techniques of the virtual lens to avoid a noisy image due to Monte Carlo integration. The spectral filters are modeled with a transparent layer of the same dimensions as the film, placed in front of the camera. The acquisition module is synchronized with the illumination stage to focus on the specimen where the illumination sheet is applied.

### System design and implementation

Our visualization system is composed of two cascaded phases: (1) virtual specimen generation, and (2) optical section rendering. In the first phase, the brain tissue model is prepared and converted into a volumetric fluorescent-tagged virtual specimen. This tissue model is extracted from a neural microcircuit composed of around 31000 neuronal morphologies and their synaptic connections. A detailed description of the microcircuit building is discussed in [41].

The neuronal morphologies are converted into three-dimensional polygonal mesh representations relying on an automated method presented by Lasserre *et al*. [42]. The spatial information of the neurons are retrieved from the microcircuit to reconstruct a tissue block composed of a group of neurons. A fluorescent tag is assigned to each neuron in this block, and finally, a GPU-based solid voxelizer is employed to convert this mesh-based block into the final virtual specimen.

After the generation of the neuronal tissue model, the rendering stage simulates the optical sectioning process of the LSFM. This stage requires a spectral rendering engine that can represent the light radiance by SPDs instead of the basic tri-stimulus RGB representation. Several rendering systems have been developed recently to meet this requirement such as LuxRender [43], Mitsuba [44] and PBRT [45]. Our system was implemented on top of PBRT for various reasons including its maintainability, the presence of good documentation describing the entire architecture of the toolkit, and its underlying implementation [25].

The light sheet model is implemented as a directional area light source with rectangular shape, uniform illumination power and single excitation wavelength. The characteristics of this virtual light sheet are defined by the profile of the light sheet waist and the wavelength of the laser unit used in the experiment.

The interaction of the synthetic light sheet and the virtual specimen is implemented in a single scattering fluorescence volume integrator that uses ray marching to evaluate the integral of the radiative transfer equation. This integrator is extended from an existing implementation of a wavelength-independent single scattering integrator that can only model elastic scattering events. Our extension accounts for both elastic and inelastic scattering events using a binary fluorescence coefficient that is equal to one if the point sampled along the path is fluorescent and zero otherwise. The fluorescence term considers the distinct properties of different fluorescent materials represented by their emission and excitation spectral profiles and their quantum yield.

The rendering of multiple fluorescent structures embedded in a single volume object requires a different design for the volume representation in PBRT. An *annotated fluorescent volume grid *is implemented to add the capability of rendering heterogeneous fluorescent structures. This grid stores at each voxel the spatial density representing the concentration of the dye and an index that refers to the intrinsic properties of this fluorescent dye.

The acquisition system is implemented with an orthographic camera that has a finite aperture lens. The focal plane of this virtual camera is synchronized with the position of the light plane. This synchronization is mandatory to obtain in-focus optical sections from the virtual specimen when the thickness of the light sheet is relatively small. A transparent two-dimensional plane with the same dimensions of image plane is added before the camera to model the emission filters in the real microscope. A spectral validation framework was integrated into PBRT to quantitatively measure the emitted power spectrum in the scene as well as the spectral radiance arriving at the film.

## Results, validation and discussion

**Figure 4:**
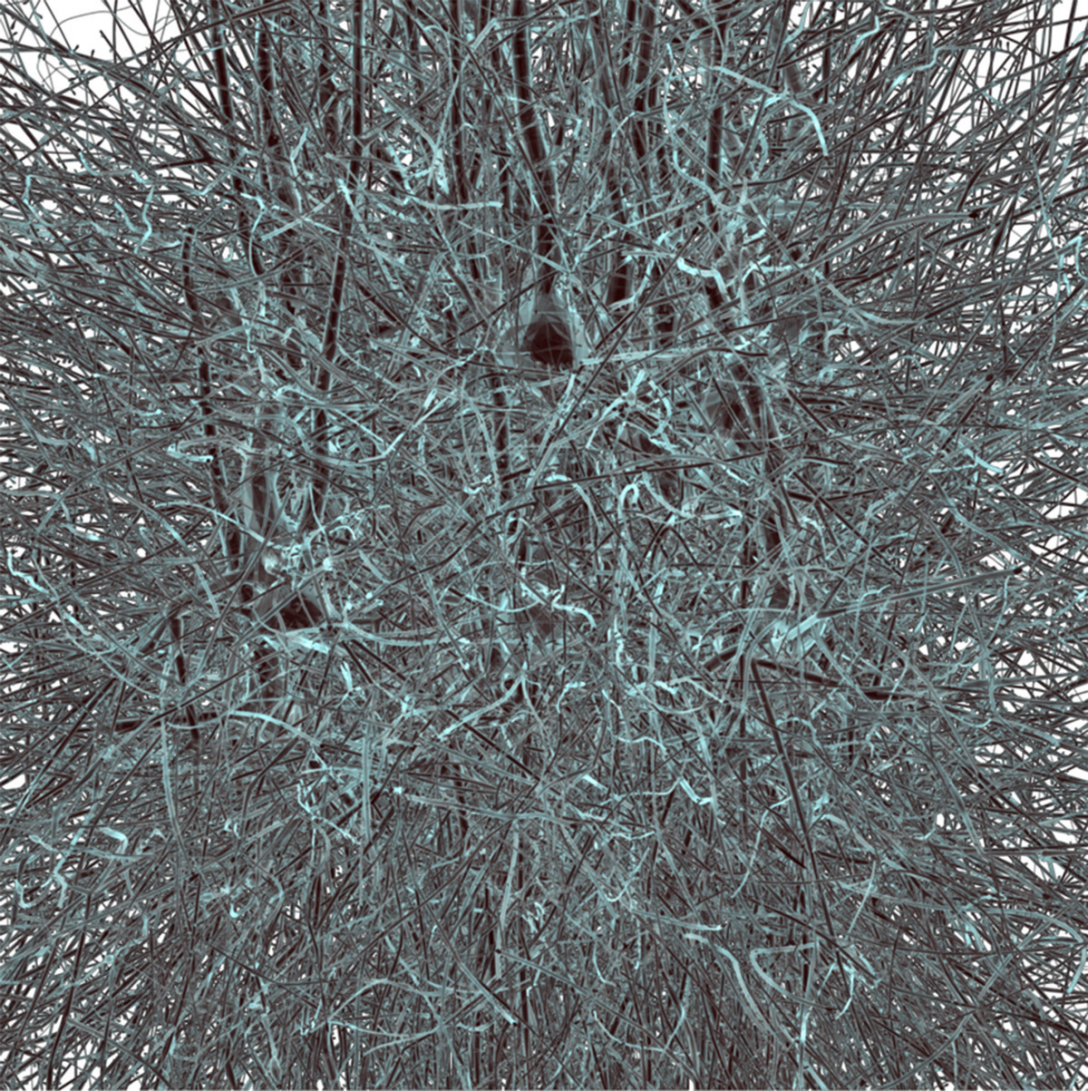
**Surface rendering of a neuronal mesh model extracted from a microcircuit reconstructed from the cortex of a young rat**. The size of this block is 100 *µ*m^3^. The virtual specimen was created by converting this model into a fluorescent tagged-volume using solid voxelization.

The results of our in silico LSFM have been demonstrated on a block of 100 *µm*^3 ^that was extracted from a microcircuit of the somatosensory cortex of a two-weeks old rat (Figure 4).

After the voxelization of this model, three virtual specimens were created and labeled with green fluorescent protein (GFP), red fluorescent protein (RFP), and cyan fluorescent protein (CFP). These fluorophores were selected due to their significant applications in neurobiology. Their intrinsic characteristics were obtained from the fluorophore database found at [46].

**Figure 5:**
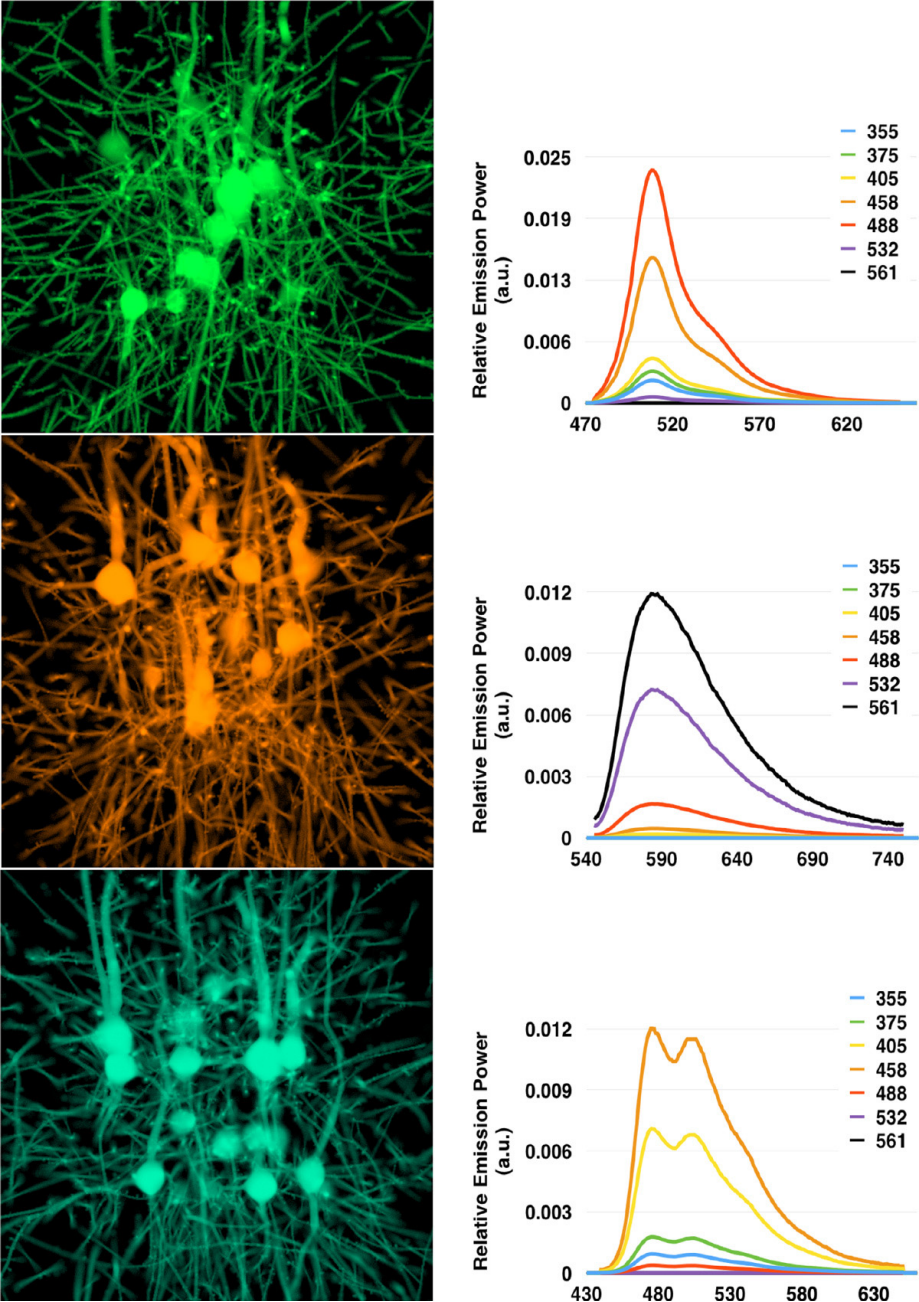
**In silico optical sectioning.** (Left) Synthetic optical sections generated from the GFP- (top), RFP- (middle), and CFP-tagged (bottom) virtual specimens excited with maximum excitation wavelength. (Right) Emission SPDs measured from the rendered images at different excitation wavelengths between 355 and 561 nm. The curves are normalized to the SPD resulting at the maximum excitation wavelength for each respective case. The x-axis of the SPDs represents the wavelength in nm.

A thin light sheet (5*µm*) was used to sample the model and generate high resolution optical sections for each virtual specimen. The current structural limitations of the tissue model do not allow to perform systematic and quantitative comparisons between a synthetic and real optical section. However, the spectral content of each image is quantitatively analysed and compared to the relative emission SPD of its corresponding fluorophore. Figure 5 shows the result of rendering different optical sections from the virtual specimens upon excitation with their corresponding maximum excitation wavelengths. The emission SPDs were computed for different excitation wavelengths between 355 and 561 nm. The detected spectral densities with our rendering workflow match the characteristic emission profiles of the three fluorophores.

**Figure 6:**
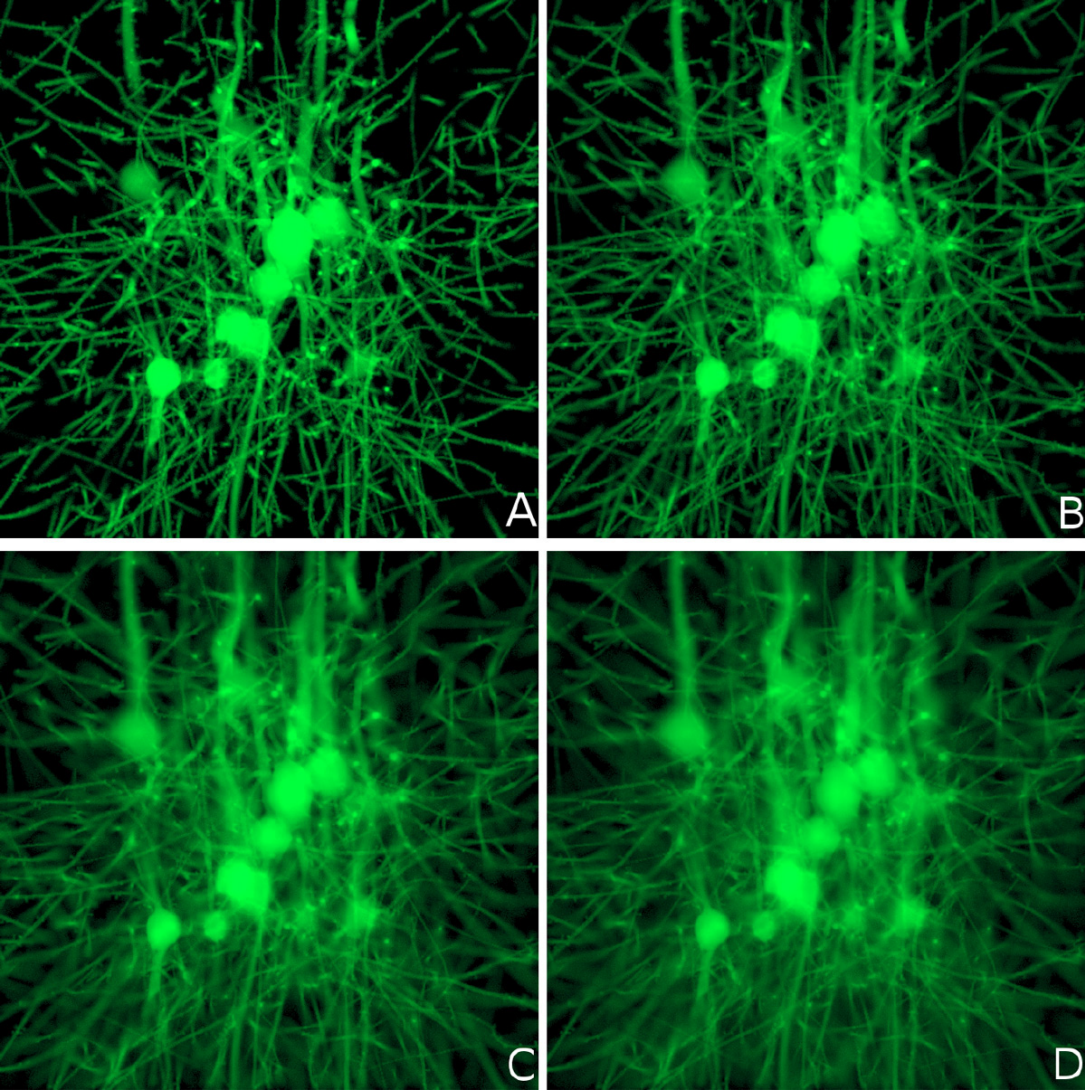
**Rendering an optical section from the GFP-tagged specimen with multiple light sheet thicknesses: (A) 5, (B) 7.5, (C) 10, and (D) 12.5 *µ*m. **The increased blur with thicker light sheets is due to the detection of out-of-focus light rays.

The axial resolution of the LSFM is inversely proportional with the thickness of the illumination sheet due to out-of-focus light contributions. The variation of the light sheet thickness is addressed to evaluate the modeling of the acquisition unit and its synchronization with the illumination one. Figure 6 shows the effect of increasing the thickness of the light sheet on the quality of the rendered optical section. The same optical section from the GFP-tagged specimen is rendered at four different light sheet thicknesses: 5, 7.5, 10, and 12.5 *µ*m respectively.

### Rendering performance

In general, the performance of rendering physically-based images depends on several factors including the sampling rate of the Monte Carlo integrator, the pixel sampling density of the image (number of sample per pixel) and the resolution of the image. High sampling rates are crucial to reduce the Monte Carlo noise. The rendering performance of our system depends on two more parameters: the depth of the optical section and the thickness of the light sheet. 32 samples per pixels were used to render our results at resolution of 1024^2^. The rendering time of the synthetic optical sections in Figures 6 and 5 varied between 103 and 120 minutes.

### Fluorescence brightness validation

**Figure 7:**
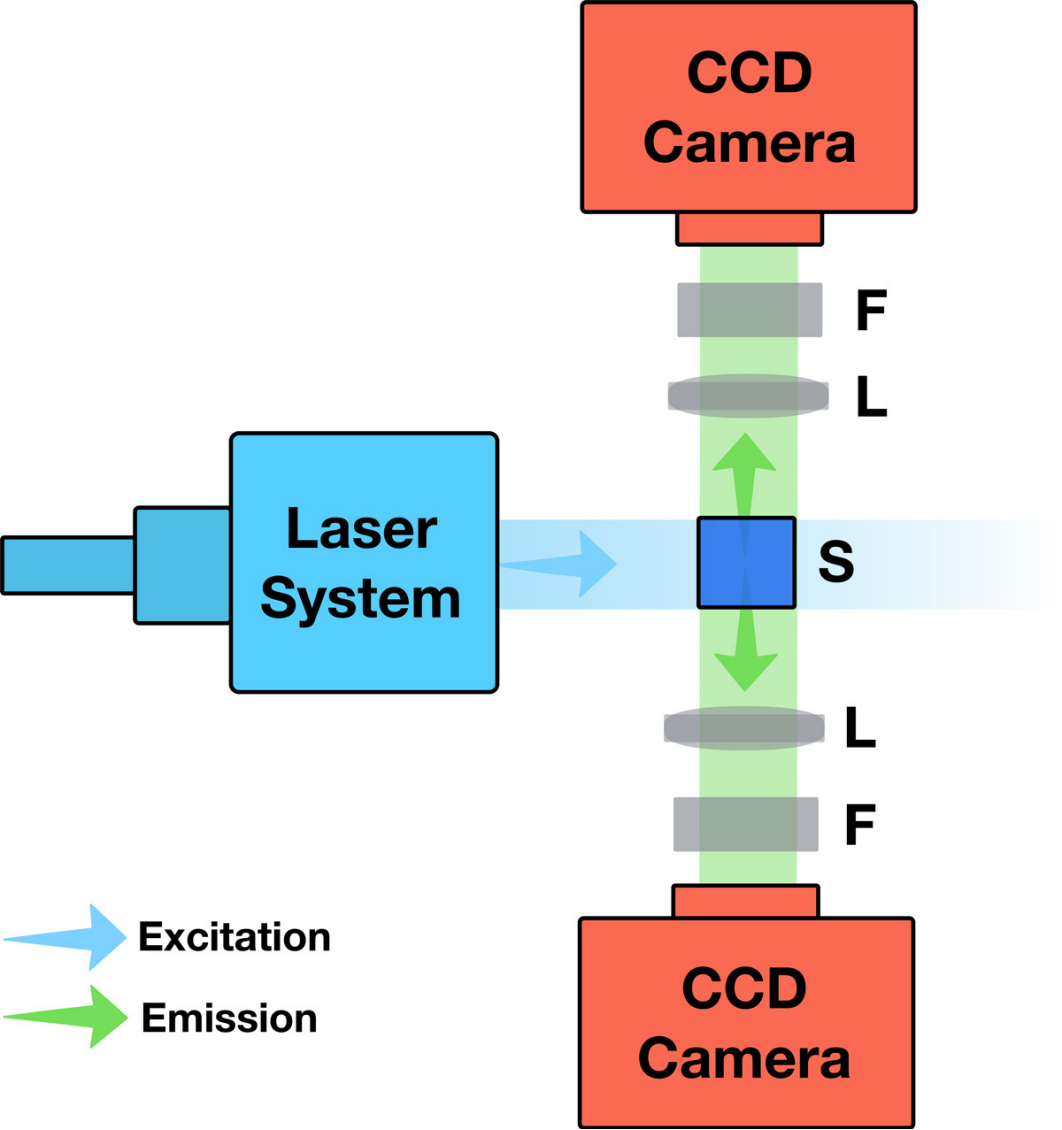
**The configuration and optical setup of the LSFM in the validation experiment**. S, F and L indicate the specimen, the filter, and the lens with finite aperture.

Although the distribution of the detected emission spectra in our in silico experiments (Figure 5) matches the exact emission profiles of the fluorescent dyes used in reality, a quantitative measure of the total number of detected photons is required to fully validate our fluorescence extension model defined by Equation (7) and to verify the integrator implementation in PBRT. This validation is feasible if the virtual specimen is represented by a homogeneous fluorescent media with defined volume geometry. To simplify this procedure, the virtual specimen was modeled by a three-dimensional homogeneous cube (2* µm*^3^) with low molecular absorption cross section (3 × 10^−16^* cm*^2^). This cube was aligned to the intersection point of the illumination and detection axes. To ensure uniform and maximum excitation of this testing volume, the dimensions of the light sheet were set to illuminate the entire cube. The emission was recorded from two opposite directions to double check the results. In theory, the recorded photon counts by the two cameras should match, but they would slightly vary due to Monte Carlo integration. The setup of our in silico LSFM for this validation experiment is illustrated in Figure 7.

The laser intensity of the exciting light sheet was varied between (1 − 10) × 10^12 ^photons. The detected photon count was integrated over the surface area of the virtual sensor and compared against the total number of fluorescent photons computed from the FBE [47].

The FBE expresses the fluorescence produced by a fluorescent molecule *F *(p) in terms of its molecular absorption cross-section *σ*, its quantum yield and the flux of the incident light beam *I*, where *F *(p) = *σ I ϕ*.

Assuming isotropic emission, the number of fluorescent photons measured at a two-dimensional plane facing any of the six planes of the cube can be computed from Equation (8), where *N *is the concentration of fluorophore in the volume, *A *is the surface area of the illumination sheet, *l *is the path length of the excitation light in the volume, *I *is the intensity of the illumination in number of photons, and *I_ϕA _*is the intensity flux density in photons per cm^2^.

(8) $$\begin{array}{c} F_{\text{FBE}}=\frac{1}{4\pi }\;I_{\phi A}\;\sigma_{s}\;N\;\phi \;A\;l\\ =\frac{1}{4\pi }\;I\;\sigma_{s}\;N\;\phi \;l \end{array}$$

The total detected number of fluorescent photons on the virtual CCD surface *F*_s _is computed from the double integral in Equation (9), where *I*(p*_S_*, λ) is the SPD measured at each point on the surface p_s_.

(9) $$F_{\text{s}}=\int_{{\text{A}}_{\text{s}}}\int_{R_{\text{v}}}I\;\left({\text{p}}_{\text{s}},\;\lambda \right)\;\text{d}\lambda \;\text{d}A_{\text{s}}$$

**Figure 8:**
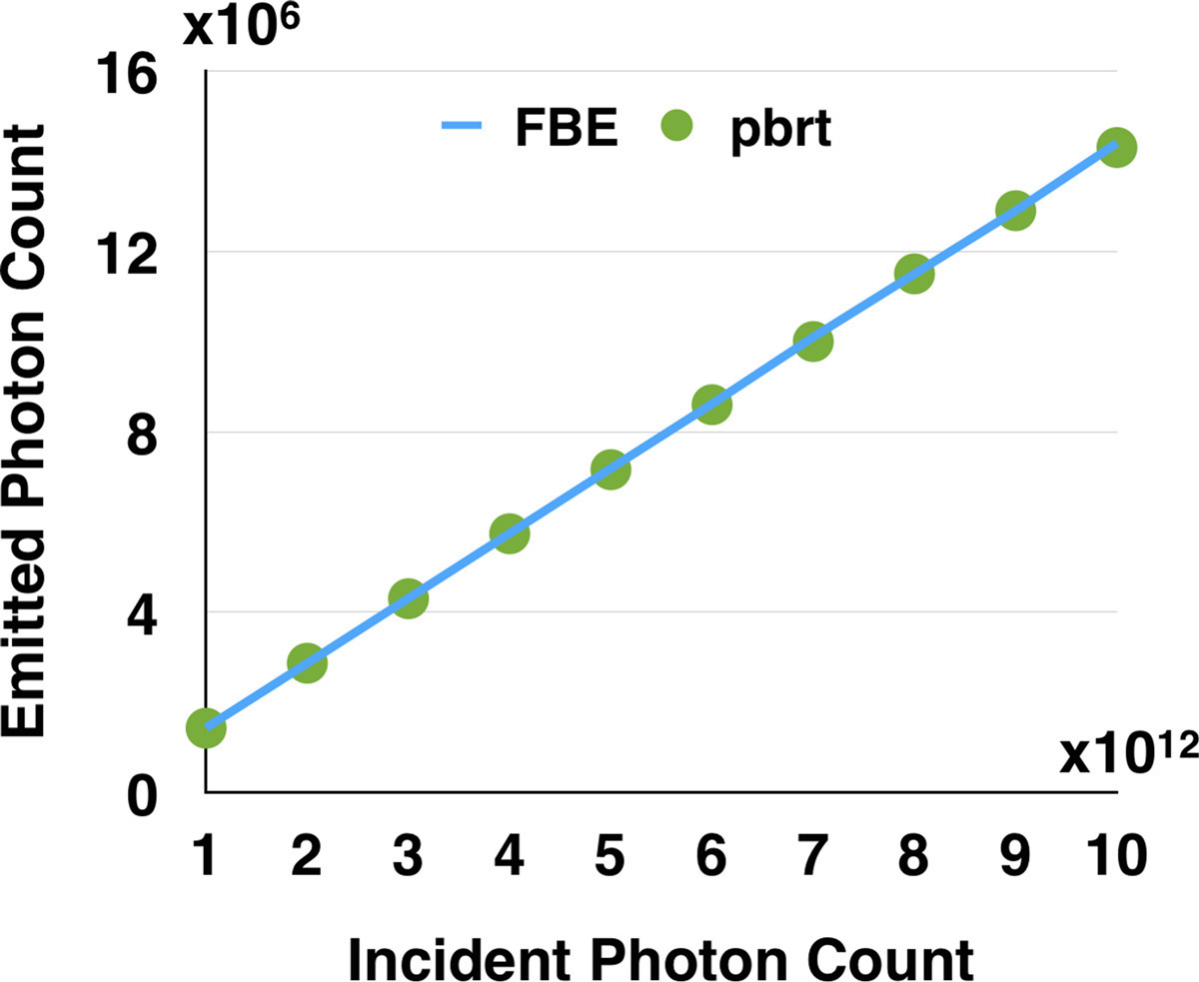
Comparison between the number of photons calculated from the FBE and those detected from the simulation in PBRT using our fluorescence model.

Figure 8 shows the validation results of our testing experiment. The total number of fluorescent photons computed from the resulting images by Equation (9) is compared against the FBE in Equation (8).

## Conclusion and future work

**Figure 9:**
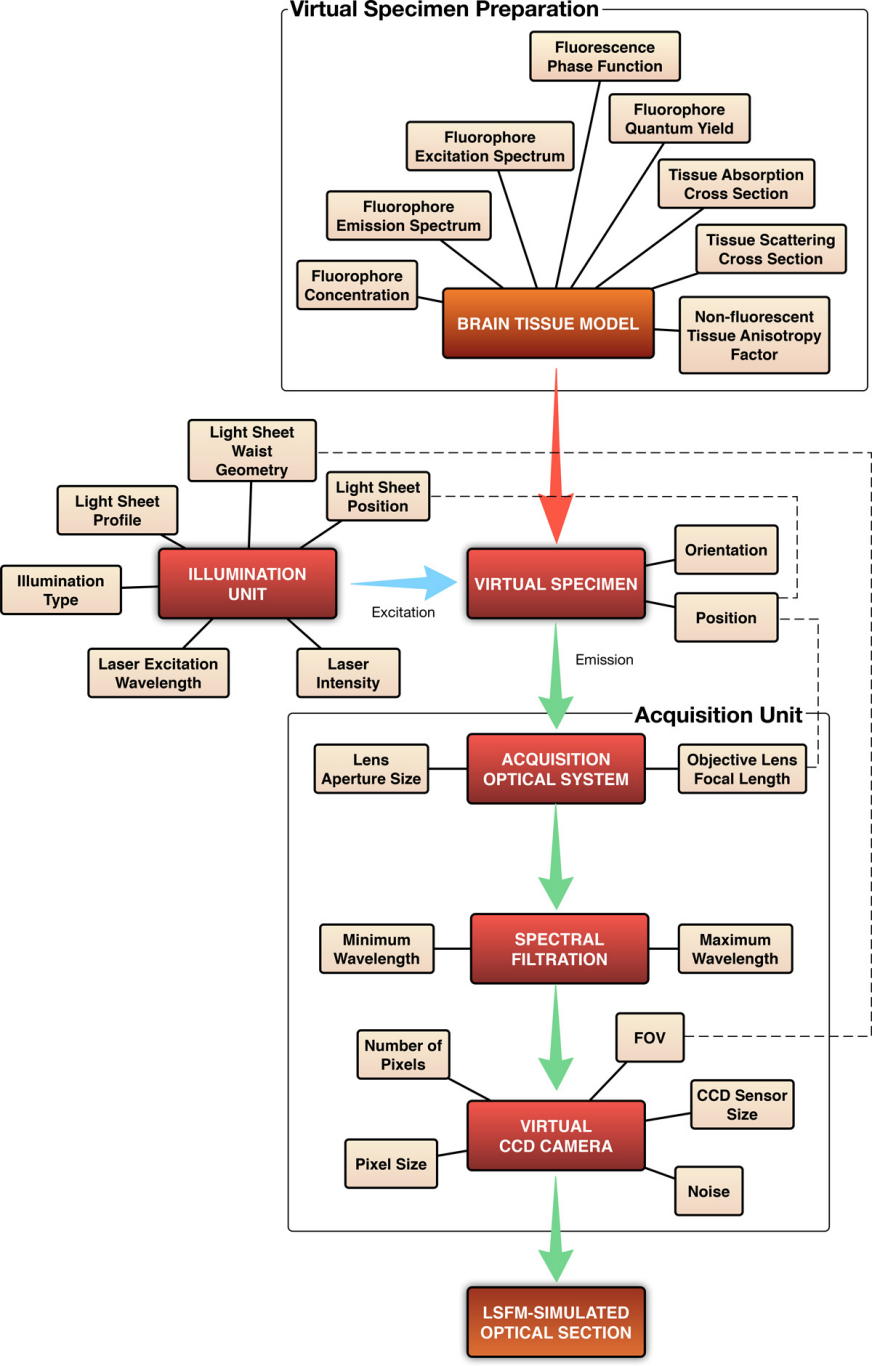
An overview of the workflow of our visualization system and its parameters.

We presented a complete computational model of the LSFM, based on geometric optics and Monte Carlo ray tracing to simulate the light transport in the pipeline of the microscope. An extension to previous fluorescence models was formulated and discussed to simulate the light interaction with fluorescent specimens. This extension was validated by comparing the results obtained with our rendering pipeline against the emission spectra of different fluorescent dyes and the brightness equation. Our LSFM simulation was used to visualize different fluorescent-tagged specimen models reconstructed from a rat cortex. The modeling aspects of the illumination and acquisition units were qualitatively analysed by varying the thickness of the illumination sheet and observing the resolution of the resulting optical sections. The code is released on GitHub at https://github.com/BlueBrain/pbrt. Figure 9 presents an overview of the entire system and shows all the simulation parameters.

We are working on extending the presented LSFM model in several directions to improve its realism. A special focus is given to simulating the missing diffraction artifacts within the ray tracer based on the reflectance model proposed in [48]. The simplified model of the light sheet will be improved to add the capability of simulating the interaction between Gaussian light sheets and wide specimen. To simulate the aberration caused by the detection objective, the current thin lens model in the acquisition system will be replaced by a realistic camera model based on the work presented by [49] and [50]. After adding these improvements, the performance of the entire microscope will be evaluated and compared to realistic microscopic images. Although the current tissue model limitations do not permit any quantitative analysis between our synthetic images and the real ones, we will use testing beads with pre-defined geometry to perform this analysis. Accelerating the performance of the system will be considered by reimplementing the entire workflow on the GPU.

## List of abbreviations used

**3D: **Three-dimensional; **CCD: **Charged Coupled Device; **CFP: **Cyan Fluorescent Protein; **DIC: **Differential Interference Contrast; **FBE: **Fluorescence Brightness Equation; **FRTE: **Full-Radiative Transfer Equation; **FOV: **Field of View; **GFP: **Green Fluorescent Protein; **GPU: **Graphics Processing Unit; **LSFM: **Light Sheet Fluorescence Microscopy; **NA: **Numeric Aperture; **PBRT: **Physically-based Rendering Toolkit; **PCM: **Phase Contrast Microscopy; **PSF: **Point Spread Function; **RFP: **Red Fluorescent Protein; **RTE: **Radiative Transfer Equation; **SPD: **Spectral Power Distribution.

## Competing interests

The authors declare that they have no competing interests.

## Authors' contributions

MA derived the mathematical model of fluorescence, implemented the rendering algorithm and drafted the manuscript. AB mentored the study and participated in the model validation and algorithm implementation. AB and SE contributed to discussions and suggestions to complete the manuscript. HM and FS supervised the project. All the authors read and approved the final manuscript.

## Declarations

This publication was supported in part by the Blue Brain Project (BBP), the Swiss National Science Foundation under Grant 200020-129525 and the King Abdullah University of Science and Technology (KAUST) through the KAUST-EPFL alliance for Neuro-Inspired High Performance Computing.

This article has been published as part of *BMC Bioinformatics *Volume 16 Supplement 11, 2015: Proceedings of the 5th Symposium on Biological Data Visualization: Part 1. The full contents of the supplement are available online at http://www.biomedcentral.com/bmcbioinformatics/supplements/16/S11
